# Rapid implementation and validation of a cold-chain free SARS-CoV-2 diagnostic testing workflow to support surge capacity

**DOI:** 10.1016/j.jcv.2020.104469

**Published:** 2020-07

**Authors:** Andrew Bosworth, Celina Whalley, Charlie Poxon, Kasun Wanigasooriya, Oliver Pickles, Erin L. Aldera, Danai Papakonstantinou, Gabriella L. Morley, Eloise M. Walker, Agnieszka E. Zielinska, Dee McLoughlin, Craig Webster, Tim Plant, Andrew Ellis, Alex Richter, I. Michael Kidd, Andrew D. Beggs

**Affiliations:** a Department of Laboratory Medicine, University Hospitals Birmingham NHS Foundation Trust, Birmingham, UK; b Regional Public Health Laboratory, Public Health England, National Infection Service, Birmingham, UK; c Surgical Research Laboratory, Insitute of Cancer & Genomic Science, University of Birmingham, Birmingham, UK; d High Containment Laboratories, University of Birmingham, Birmingham, UK; e Clinical Immunology Service, University of Birmingham, Birmingham, UK

**Keywords:** VIASURE qRT-PCR, COVID-19, SARS-CoV-2, Coronavirus, Rapid response, West Midlands, Birmingham

## Abstract

**Background:**

In January 2020 reports of unidentified severe respiratory illness were described in Wuhan, China. A rapid expansion in cases affecting most countries around the globe led to major changes in the way people live their daily lives. In the United Kingdom, the Department of Health and Social Care directed healthcare providers to establish additional resources to manage the anticipated surge in cases that could overwhelm the health services. A priority area was testing for SARS-CoV-2 RNA and its detection by qualitative RT-PCR.

**Design:**

A laboratory workflow twinning research environment with clinical laboratory capabilities was implemented and validated in the University of Birmingham within 4 days of the project initiation. The diagnostic capability was centred on an IVD CE-marked RT-PCR kit and designed to provide surge capacity to the nearby Queen Elizabeth Hospital. The service was initially tasked with testing healthcare workers (HCW) using throat swabs, and subsequently the process investigated the utility of using saliva as an alternative sample type.

**Results:**

Between the 8th April 2020 and the 30th April 2020, the laboratory tested a total of 1282 HCW for SARS-CoV-2 RNA in throat swabs. RNA was detected in 54 % of those who reported symptoms compatible with COVID-19, but in only 4% who were asymptomatic.

**Conclusion:**

This capability was established rapidly and utilised a cold-chain free methodology, applicable to a wide range of settings, and which can provide surge capacity and support to clinical laboratories facing increasing pressure during periods of national crisis.

## 1 Background

SARS-CoV-2 is an emerging sarbecovirus, closely related to SARS-CoV-1 that emerged in China in 2004 [1]. This new strain was first described in January 2020 as a cause of a novel severe respiratory illness now known as COVID-19. As of the 3rd May 3,356,205 confirmed cases were reported globally with 238,730 deaths, affecting 215 countries around the world [2]. The rapid development of the pandemic highlights the need to speedily assemble the capability to support healthcare services in a variety of international settings. Responsive capacity building was part of the United Kingdom’s strategy to rapidly scale up testing for SARS-CoV-2 RNA in respiratory samples using qRT-PCR. A directive from the Department of Health and Social Care, and National Health Service Improvement (NHSI) instructed NHS hospitals to identify the means to increase testing capacity [3]. Accordingly, the University of Birmingham (UoB) was included in an extended laboratory network to provide additional capacity to the existing diagnostic laboratories in the West Midlands region, particularly to support healthcare worker testing. Rapid technology and knowledge transfer combined with a partnership between a UKAS-accredited clinical service and academic research infrastructure reconfigured the laboratories into a molecular diagnostic service capable of providing quality assured testing using a verified commercial test. External support was provided by Public Health England clinical virologists in an advisory role.

This manuscript describes the rapid implementation and validation of the process to support healthcare worker testing in the West Midlands at the University of Birmingham.

## 2 Study design and materials

Initially, 120 specimens were tested by both the SARS-CoV-2 testing process at the University and at the Queen Elizabeth Hospital to verify the assay and validate the diagnostic process. This total consisted of 94 residual RNA extracts anonymised at source, and 26 respiratory swabs collected from in-patients giving informed consent to provide 2 swabs to assist with improving diagnostics.

Secondly, a total of 1283 nose & throat swabs were collected from volunteer healthcare workers (HCW) giving informed consent for their samples to be tested for SARS-2-CoV. Of these, 258 specimens were self-taken, and 1025 collected by research nurses in the Institute of Translational Medicine.

Samples were received and logged at Containment Level 2 (CL-2) before safe transfer to Advisory Committee on Dangerous Pathogens (ACDP) Containment Level 3 (CL-3) facilities, where sample inactivation [5] was performed with buffer ATL (Qiagen). Samples were processed further at CL-2 using the RNEasy Mini Kit reagents (Qiagen). qRT-PCR testing for SARS-CoV-2 RNA was performed using the VIASURE qRT-PCR detection test for SARS-CoV-2 (CerTest; Prolabs). The VIASURE test is a multiplex of three targets: the viral N and ORF1ab genes (China-CDC) [4], and a synthetic internal control to detect assay inhibition. Lyophilized reaction reagents were reconstituted, 15 μl of master mix used per reaction with the addition of 5 μl sample RNA. Thermocycling was performed on either ABI 7500 or QuantStudio 5 instruments. All preliminary data analysis from qRT-PCR analyses was performed using the Thermo Cloud software (Thermo Scientific), with automated threshold and baseline setting followed by careful manual inspection of individual amplification curves by two senior experienced operators. Final interpretation of the RT-PCR assay results was as specified in the manufacturer instructions for use (IFU). Confirmatory testing on indeterminate results was performed with E gene assay [4]. These results were transmitted electronically to the UKAS-accredited service, for reporting to the NHS Trust and Public Health England surveillance systems.

## 3 Study results

**Fig. 1:**
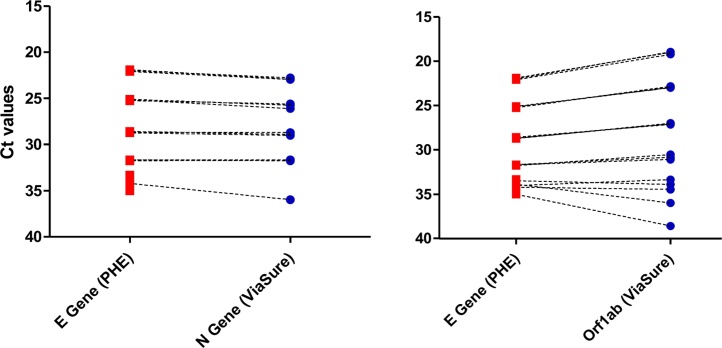
Linear dilution series was performed on extracted RNA derived from pooled throat swabs with detectable SARS-CoV-2 RNA. Data from linear dilution series in the VIASURE in triplicate was compared to the results obtained with the E-gene (WHO) assay in a pairwise comparison.

To investigate the analytical sensitivity of the VIASURE qRT-PCR, it was compared to the analytical sensitivity of the SARS-2-CoV ‘E’ gene assay described by the WHO [4], and which is a more widely characterised tests (Table 1). The VIASURE Orf1ab detection appears to be an estimated 10-fold less sensitive than the E gene assay, but 10-fold more sensitive than the RdRp gene. The VIASURE N gene assay appeared to be of comparable sensitivity to the RdRp gene target (Fig. 1).

**Table 1:** Relative analytical sensitivity between the VIASURE qRT-PCR assay (Orf1ab and N gene) and the WHO E gene and RdRp assay. Proportion of replicates detection in a 10 fold linear dilution series from 10e-1 to 10e-8, the mean Ct value obtained for each dilution in each of the assays tested is also given. N.D. denotes Not Detected. Underlined are the last dilutions showing 100 % detection rates in the replicates. Each dilution was performed in duplicate and each duplicate tested in triplicate.

		Replicates Detected				Mean Ct Values			
		E Gene	RdRp	Orf1ab	N Gene	E Gene	RdRp	Orf1ab	N Gene
Linear Dilution Series (10 fold)	1.00E-01	100 %	100 %	100 %	100 %	20.4	24.3	19.1	22.9
	1.00E-02	100 %	100 %	100 %	100 %	23.5	27.3	22.9	25.8
	1.00E-03	100 %	100 %	100 %	100 %	27.2	30.9	27.1	28.9
	1.00E-04	100 %	100 %	100 %	100 %	30.6	34.5	30.8	31.7
	1.00E-05	100 %	66 %	100 %	66 %	34.5	36.5	33.9	36.0
	1.00E-06	66 %	N.D.	66 %	N.D.	38.2	N.D.	37.3	N.D.
	1.00E-07	33%	N.D.	N.D.	N.D.	39.1	N.D.	N.D.	N.D.
	1.00E-08	N.D.	N.D.	N.D.	N.D.	N.D.	N.D.	N.D.	N.D.

**Fig. 2:**
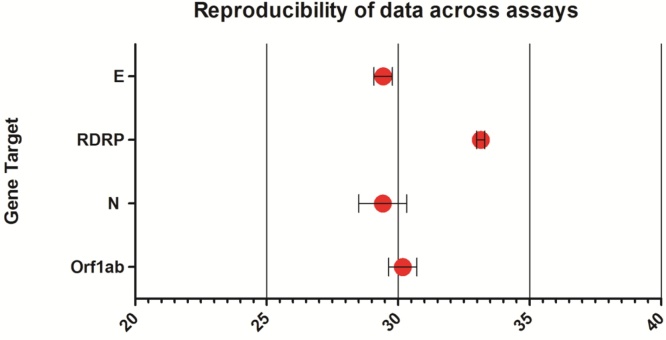
Analysis of reproducibility testing data using positive control specimens (n = 10) tested in triplicate on the E gene, RDRP WHO assays compared to the N and Orf1ab VIASURE targets. Standard deviation is calculated for variation from the mean. The mean is given as a large red marker on the chart. The X-axis indicates Ct values obtained in each assay for comparison.

Comparative testing against other validated assays was performed using in-house control material derived from pooled patient specimens where RNA from SARS-CoV-2 had been detected. Samples were tested on WHO RdRp and E gene assays in parallel with the VIASURE qRT-PCR assay. The mean Ct value obtained with the E gene (WHO), the N gene and Orf1ab gene assays (VIASURE) were not significantly different by repeated measures ANOVA. However, analysis demonstrated that RdRp was significantly different from E gene, N-gene and Orf1ab gene (p=<0.0001), with an average deviation in Ct value of +5.4 Ct, indicating a significant difference in assay sensitivity (Fig. 2).

**Fig. 3:**
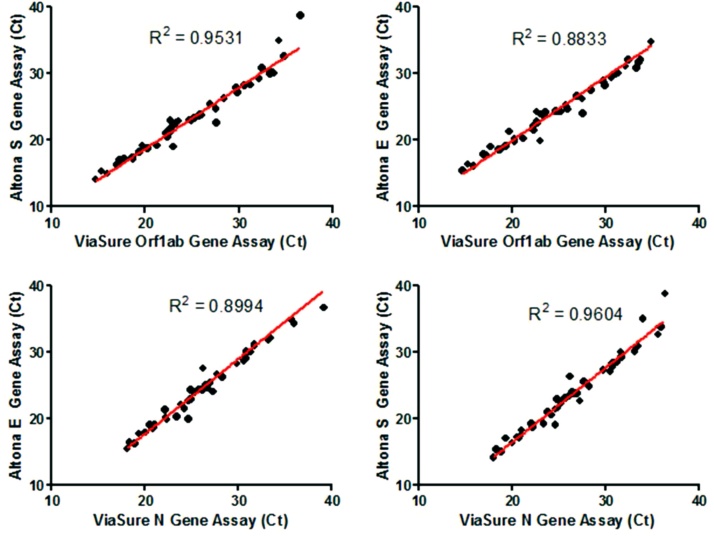
Pearson correlation analysis of results obtained from the N gene assay and the Altona assay performed at the Queen Elizabeth Hospital, calculated R^2^ values are displayed on each chart, and the trend displayed as a red line.

Gaining experience of qRT-PCR testing and reproducibility of the workflow was initially assessed by testing (a) a panel of residual RNA preparations from the Queen Elizabeth Hospital which had been extracted and previously tested on the Altona commercial qRT-PCR assay (n = 94) or (b) original respiratory samples collected from patients the same day and tested on an Abbott m2000 commercial qRT-PCR assay (n = 26). Results of these comparisons are shown in Table 2. The Ct values obtained for each assay were analysed by Pearson correlation analysis in a pairwise fashion, indicating a strong correlation in all comparisons (R^2^ = >0.88; Fig. 3).

**Table 2:** Clinical sensitivity of the VIASURE qRT-PCR Kit for detection of SARS-CoV-2 as determined in comparative analysis in the University of Birmingham and Queen Elizabeth Hospital. Two commercial assays were used for comparison, the Altona qRT-PCR assay (n = 94) and the Abbott m2000 qRT-PCR assay (n = 26).

		Queen Elizabeth Hospital RT-PCRs					
		Altona Assay			Abbott Assay		
		Positive	Negative	Total	Positive	Negative	Total
University of Birmingham VIASURE qRT-PCR	Positive	**48**	**1**	49	**10**	**0**	10
	Negative	**0**	**42**	42	**0**	**16**	16
	Indeterminate	**0**	**3**	3	**0**	**0**	0
	Totals	48	46	94	10	16	26

In the comparison of the 94 RNA extracts tested by VIASURE and Altona, the majority of positive and negative results agreed between the two tests. Importantly, there were no Altona positive results that were found negative by VIASURE. Three Altona negative samples had equivocal results on VIASURE, where the ORF1ab target was negative but N gene target detected at Ct < 38; albeit still relative high Cts (at 36, 33, and 36).

In the second analysis of 26 clinical samples, the end-to-end process of testing from fresh respiratory specimens was compared with that at QEH. By coincidence, the QEH Pathology testing method had changed from Altona to Abbott m2000, an assay which has acknowledged greater analytical sensitivity than the Altona. Nevertheless, the results of the direct comparison showed that there was 100 % concordance between positive and negative results obtained by VIASURE and the Abbott test (Table 2). Combining the results and experience of performing the VIASURE test in a full diagnostic process, with further adjustments to reduce the chances of contamination, the VIASURE test was considered as verified for use.

Accordingly, the VIASURE test was utilized in the analysis of symptomatic and asymptomatic HCW. Throat swab samples were taken from 1283 subjects and tested between the 8th April and the 30th April, with an overall SARS-2-CoV detection rate of 53/1230. A detection frequency of 15 % (37/258) was found in cases where symptoms were in keeping with COVID-19 diagnosis. Sixteen of 1025 (1.6 %) HCW with no compatible symptoms and defined as asymptomatic had detectable levels of viral RNA in their throat swabs.

Saliva was collected from 525 of the same HCW cohort as part of a prevalence study of asymptomatic viral shedding. Fifteen oral fluid samples from subjects with positive results by throat swab, were tested using the VIASURE, of which five were positive. The Ct values were lower (by approximately 3–5 Ct) compared to results on the subject’s throat swab, suggesting a higher viral load in saliva. Interestingly, follow-up of these asymptomatic HCW whose saliva specimens were positive indicated that they later developed symptoms compatible with COVID-19 disease. It is unclear however whether patients were truly asymptomatic at the time of sampling, or possibly overlooked mild symptoms; as they were not formally clinically assessed. Therefore, saliva may represent a specimen type that can provide a more unambiguous indicator of potential infectivity, removing the equivocality of low-level positive throat swab results.

The capacity to rapidly deploy extra laboratory capabilities to support surges in diagnostic testing workload, or to support the development of testing infrastructure in rural and remote areas, will be essential to controlling the current SARS-CoV-2 pandemic.

## Declaration of Competing Interest

The authors declare no conflict of interest.

## Funding

The work described in this manuscript was funded by National Health Service Improvement (NHSI) with contributions directly from the University Hospitals Birmingham NHS Foundation Trust, core funding from the University of Birmingham, and staff from Public Health England participated in the establishment of the service. ADB is currently supported by a Cancer Research UK Advanced Clinician Scientist award (C31641/A23923) and his laboratory is supported by a Cancer Research UK Centre Award and Experimental Cancer Medicine Centre Award (C11497/A25127).

## References

[1] Luk HKH Li X., Fung J., Lau S.K.P., Woo P.C.Y. (2019). Molecular epidemiology, evolution and phylogeny of SARS coronavirus. Infect. Genet. Evol..

[2] Dong E., Du H., Gardner L. (2020). An interactive web-based dashboard to track COVID-19 in real time. Lancet Infect. Dis..

[3] Coronavirus (COVID-19): scaling up our testing programmes [https://www.gov.uk/government/publications/coronavirus-covid-19-scaling-up-testing-programmes/coronavirus-covid-19-scaling-up-our-testing-programmes].

[4] Corman V.M., Landt O., Kaiser M., Molenkamp R., Meijer A., Chu D.K., Bleicker T., Brünink S., Schneider J., Schmidt M.L. (2020). Detection of 2019 novel coronavirus (2019-nCoV) by real-time RT-PCR. Eurosurveillance.

